# Dyschromatosis Symmetrica Hereditaria of Late Onset?

**DOI:** 10.1155/2014/639537

**Published:** 2014-02-04

**Authors:** Caroline Balvedi Gaiewski, Sergio Zuneda Serafini, Betina Werner, Janyana M. D. Deonizio

**Affiliations:** ^1^Dermatology Department, Federal University of Parana, 80530-905 Curitiba, PR, Brazil; ^2^Pathology Department, Federal University of Parana, 80530-905 Curitiba, PR, Brazil

## Abstract

Dyschromatosis symmetrica hereditaria (DSH), also known as reticulated acropigmentation of Dohi, is an autosomal dominant disease with high penetrance, characterized by hypo- and hyperpigmented macules of varying sizes on the dorsal of the extremities with reticulated pattern. This paper presents a female patient with typical dermatological lesions, but only diagnosed in adulthood. It is necessary to perform differential diagnosis with other pigmentary disorders. This entity is not very common in South America, and the vast majority of cases were described in Japanese population. Since it is a benign disease, it is important to be aware of this diagnosis in order to establish the correct conduct for these patients.

## 1. Introduction

Dyschromatoses are characterized by the presence of hyper- and hypopigmented macules arranged in a reticular pattern. Dyschromatosis symmetric hereditaria (DSH) is a rare genodermatosis, autosomal dominant with high penetrance, with some sporadic reported cases. It is often reported in Japanese patients, but there have been also cases in India, Europe, and South America [1, 2]. After the literature review in PubMed database, this is the fourth case reported in Brazil [3, 4].

## 2. Case Report

**Figure 1:**
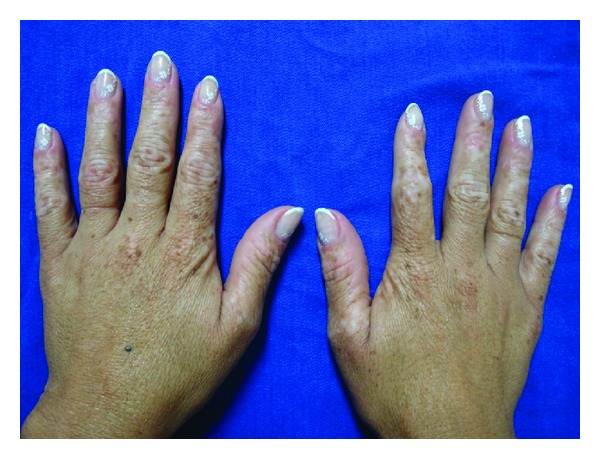
Hyper- and hypopigmented macules distributed symmetrically on the dorsum of the hands.

**Figure 2:**
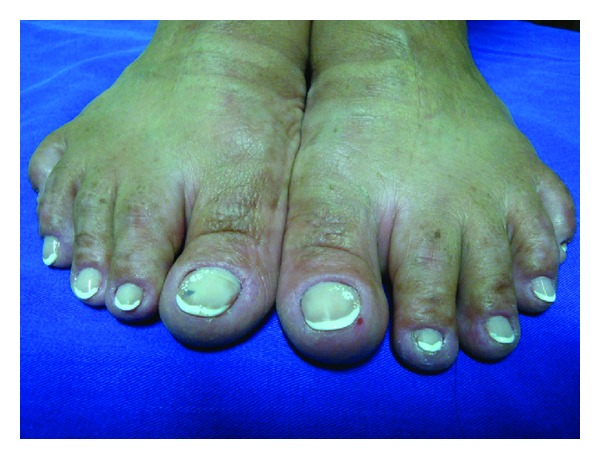
Hyper- and hypopigmented macules distributed symmetrically on the dorsum of the feet.

**Figure 3:**
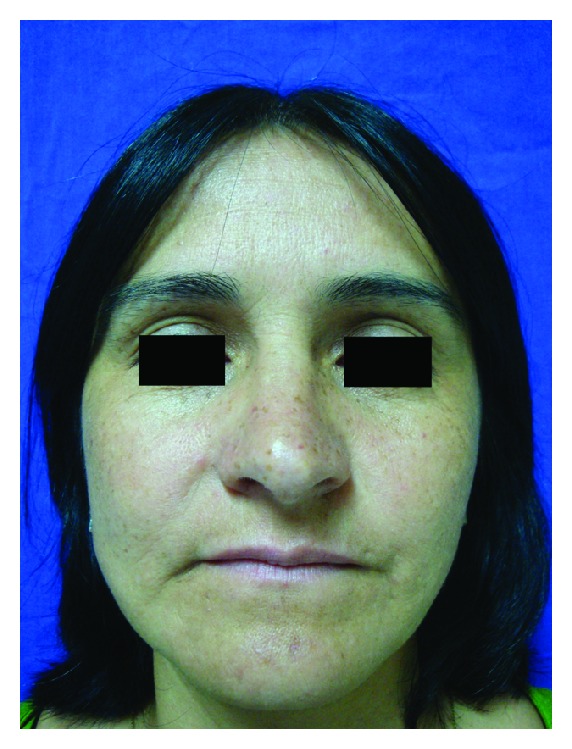
Multiple freckle-like pigmented macules on the face.

A 40-year-old female patient presented with multiple small hyperchromic and hypochromic macules distributed symmetrically on the dorsum of the hands and feet (Figures 1 and 2). She noted these lesions when she was 26 years old, and they were asymptomatic. Moreover multiple freckle-like pigmented macules were also noted on her face (Figure 3). She denies other comorbidities. Investigation for autoimmune disease and other laboratory findings were negative. Family history was positive with a nephew with similar lesions.

**Figure 4:**
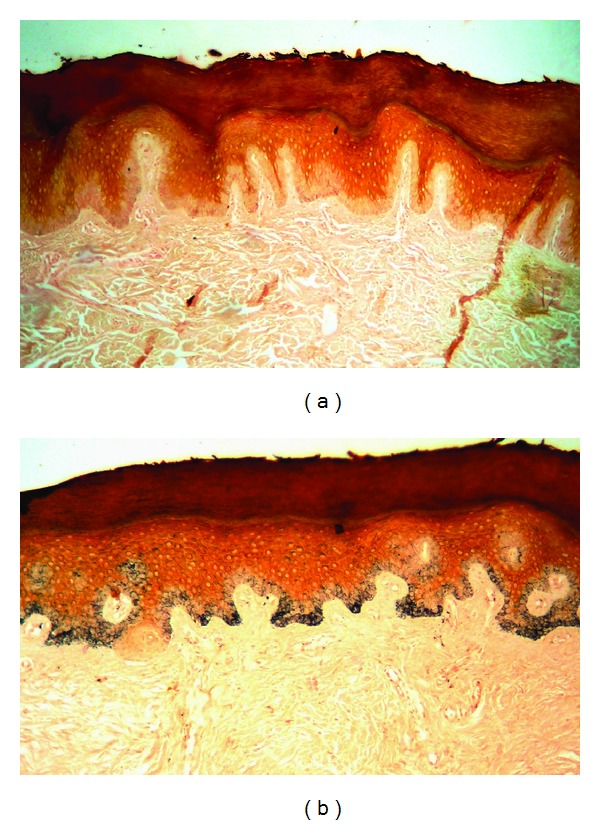
(a) Histology from hypochromic lesion showing decrease, with almost absence of melanin. (b) Histology from hyperchromic region showing intense amount of pigment (Fontana-Masson, 100X, 2X).

Two different biopsies were performed from hypochromic and hyperchromic areas. From hypochromic lesion, histology demonstrated a compact stratum corneum and discrete acanthosis. Using Fontana Masson staining, hypochromic lesion demonstrated marked decrease, with almost absence, of melanin. On the other hand, the hyperchromic lesion demonstrated an intense pigmentation (Figures 4(a) and 4(b)). Those findings were compatible with the clinical hypothesis of DSH.

## 3. Discussion

DSH is a genodermatosis characterized by multiple small hypo- and hyperpigmented macules, with irregular size and shape, symmetrically distributed on the backs of the hands and feet. Some patients also show freckle-like macules on the face. Palms, plants, and mucous membranes are free from the disorder [5]. Lesions appear in childhood, usually before the age of six, remaining during life without any color or distribution changes after stabilization in adolescence. In general, the involvement is limited to skin, without systemic involvement or evidence of photosensitivity. However, there are isolated reports of association with neurofibromatosis type I, thalassemia, polydactyly, and torsion dystonia [6].

Since 2003, a genetic mutation has been identified on chromosome IqII-Iq2I as responsible for the production and distribution changes of melanin. Miyamura et al. were the first to identify heterozygous mutations of the gene *DSRAD* or ADAR1, responsible for codifying the double-stranded adenosine deaminase specific RNA, as the cause of DSH [1, 6]. Despite more than 90 different mutations in the *DSRAD* gene having been described in the literature, it is still uncertain how these changes can cause the same phenotype [7].

Histologically, in hypopigmented areas, the deposit of melanin is scarce, in contrast to the areas of hyperpigmentation that contain melanocytes with an increased metabolic activity and high concentration of melanosomes [6]. The number of melanocytes in the areas of hypopigmentation is diminished compared to individuals without the disease [1].

This disease must be differentiated from other pigmentary disorders such as reticulate acropigmentation of Kitamura, which is marked by presence of atrophy and absence of hypopigmented lesions; Dowling-Degos disease by reticulate hyperpigmentation in the body's folds, accompanied by comedogenic lesions on back and neck, and depressed or pitted scars; initial cases of xeroderma pigmentosum distinguished by the development of more serious symptoms of xerosis, atrophy, telangiectasia, and tumors in photo-exposed areas; dyschromatosis universalis hereditaria with predominating lesions in trunk starting in childhood; vitiligo with repigmentation areas which can simulate hyperchromic macules of DSH but have perifollicular distribution [1, 2, 6, 8].

Interestingly the age of onset of DSH is 4.4 years old in average, which differs from our case (26 years old). Dowling-Degos disease and Galli-Galli disease are characterized by a later onset (24.5 and 45.6, resp.) and may be considered as a differential diagnosis [2]. Nevertheless, we favor the diagnosis of DSH once Dowling-Degos disease presents with reticulate hyperpigmented macules in the flexures and large body folds. Galli-Galli disease is characterized by reticulated pigmented and hyperkeratotic erythematous macules and papules, located in the same areas of Dowling-Degos disease, but has a very peculiar histology demonstrating moderate-to-severe acantholysis of the suprabasal epidermis, which was not observed in our case. Adult-onset dyschromatosis often is secondary to chemicals, drugs, physical agents, cutaneous lupus erythematous, or infection [9]. We excluded those causes by history and laboratory investigation. We believe that this is a true case of DSH since the patient has very typical clinical findings and positive family history of similar lesions. It is possible that lesions have been unnoticed until adulthood.

No treatment is effective for this genodermatosis [6]. The exact frequency of the DSH is unknown since the main changes are confined to the skin with no systemic involvement, and many cases remain unreported. Despite the several reports and genetic studies from East Asian countries, the DSH is a very rare diagnosis in Brazil. We report this case due to its rare occurrence in South America and in view of the need of proper diagnosis and guidance for these patients.

## References

[1] Hayashi M, Suzuki T (2013). Dyschromatosis symmetrica hereditaria. *The Journal of Dermatology*.

[2] Müller CS, Tremezaygues L, Pföhler C, Vogt T (2012). The spectrum of reticulate pigment disorders of the skin revisited. *European Journal of Dermatology*.

[3] Froes GC, Pereira LB, Rocha VB (2009). Case for diagnosis. *The Journal Brazilian Annals of Dermatology*.

[4] Fernandes NC, Andrade LR (2010). Case for diagnosis. *Anais Brasileiros de Dermatologia*.

[5] Vachiramon V, Thadanipon K, Chanprapaph K (2011). Infancy- and childhood-onset dyschromatoses. *Clinical and Experimental Dermatology*.

[6] Consigli J, Gómez Zanni MS, Ragazzini L, Danielo C (2010). Dyschromatosis symmetrica hereditaria: report of a sporadic case. *International Journal of Dermatology*.

[7] Liu Y, Liu F, Wang X (2012). Two novel frameshift mutations of the DSRAD gene in Chinese pedigrees with dyschromatosis symmetrica hereditaria. *International Journal of Dermatology*.

[8] Mohana D, Verma U, Amar AJ, Choudhary RKP (2012). Reticulate acropigmentation of dohi: a case report with insight into genodermatoses with mottled pigmentation. *Indian Journal of Dermatology*.

[9] Vachiramon V, Thadanipon K, Rattanakaemakorn P (2012). Adult-onset dyschromatoses. *Clinical and Experimental Dermatology*.

